# Determination of phytochemical quality of leaves *Vaccinium vitis-idaea* L. and *Vaccinium myrtillus* L. from the polluted and non-polluted areas

**DOI:** 10.1007/s10661-024-13157-1

**Published:** 2024-10-30

**Authors:** Veronika Petruľová, Miriam Bačkorová

**Affiliations:** 1 Department of Botany, Institute of Biology and Ecology, Faculty of Science, Pavol Jozef Šafárik University, Mánesova 23, Košice, 041 67 Slovakia; 2 Present Address: Department of Pharmaceutical Technology, Pharmacognosy and Botany, University of Veterinary Medicine and Pharmacy in Košice, Košice, 041 81 Slovakia

**Keywords:** Lingonberry, Bilberry, Scanning electron microscopy, Chromatography, Pollution

## Abstract

Elemental composition of *Vaccinium myrtillus* L*.* and *Vaccinium vitis-idaea* L. has not been determined yet in detail. In our study, a scanning electron microscope coupled with an energy dispersive X-ray analyser (SEM–EDX) ensured the determination of 15 elements in the leaves of *Vaccinium* sp. growing in the control and the mine polluted locality. The soil elemental analyses showed a higher content of 11 elements from all determined in the mine-influenced samples. Elemental analyses of the control leaves showed the highest contents of all determined elements for *V. vitis-idaea*, except for carbon. The impact of pollution on *V. myrtillus* leaves caused significant increase in oxygen, natrium, magnesium, sulphur, chlorine, potassium and calcium content. The contents of carbon, nitrogen and silicon decreased substantially. In the case of the *V. vitis-idaea* leaves, the content of most of elements reduced, and in the case of six elements, even significantly. A significant increase was recorded for carbon, iron and copper. The effect of the environment has influenced the production of phytochemicals, assessed by HPLC–DAD. The production of flavonoids (hyperoside, isoquercetin) increased significantly in *V. myrtillus* and decreased to a detectable minimum in *V. vitis-idaea.* In the case of chlorogenic acid, arbutin and hydroquinone, their levels changed minimally in *V. myrtillus*, but significantly more in the *V. vitis-idaea* leaves. The importance of elemental analyses lies in detecting the presence of toxic elements or their harmful concentrations in plants that are the source of food or dietary supplements.

## Introduction

*Vaccinium* sp. plants (*V. myrtillus* L. and *V. vitis-idaea* L.), from the Ericaceae family, belong to interesting medicinal plants due to their phytochemical production, which includes flavonoids (especially kaempferol and quercetin, and their derivatives), hydroquinones (e.g. arbutin), anthocyanins (e.g. delphinidin) and phenylpropanoids (e.g. hydroxycinnamic acids, chlorogenic acid). Thanks to their content, *Vaccinium* plants are a rich source of antioxidants and are effective in the treatment of many diseases, e.g. urinary tract infections, retinal diseases, digestive problems and diabetes mellitus (Ieri et al., 2013; Sadowska et al., 2014; Ștefănescu et al., 2019).

From an ecological point of view, *Vaccinium* sp. are oligotrophic plants with a preference for the ammonium nitrogen (N) and low phosphorus (P), potassium (K), calcium (Ca) and magnesium (Mg) requirements. Thanks to reactivity of their enzymatic and non-enzymatic antioxidant systems (e.g. guaiacol peroxidase, superoxide dismutase, proline, non-protein thiols, glutathione, ascorbic acid) in connection with accumulation of elements in the plant parts, especially in the roots, they can tolerate an environmental excess of elemental nutrients or heavy metals (Kandziora-Ciupa et al., 2017; Zhang et al., 2023). Therefore, their elemental composition and phytochemical content can change (Uhlig & Junttila, 2001).

The scanning electron microscope connected with energy dispersive X-ray analyser (SEM–EDX) is generally used to determine the microstructure shapes or elemental identification with quantitative compositional information. The method measures objects on the surface, but X-rays may reach a depth of up to 100 nm (Filipiak-Szok et al., 2015), so that it can provide valuable information on the elemental composition of the studied material. Different materials, like archaeological excavations (Shillito et al., 2009), micro-residues (Lynch & Miotti, 2017), microplastics (Gniadek & Dabrowska, 2019), animal cell tissues (Zhu et al., 2023) and food samples (Filipiak-Szok et al., 2015), were analysed in this way. For plant materials, SEM–EDX has been mainly used for anatomical studies or partial elemental determinations of microstructures (e.g. phytoliths and metalloids), which have already been published previously (Di Baccio et al., 2009; He et al., 2014; Lombi et al., 2011; Nakphlaiphan et al., 2016; Rossini Oliva et al., 2009).

Up to now, the determination of chemical elements in *Vaccinium* plants has been carried out by various methods. For example, Dróżdż et al. (2017) determined 13 metal elements in the fruits of wild-growing blueberries by inductively coupled plasma (ICP OES) using a Thermo Scientific spectrometer. Karlson et al. (2018) determined twelve biologically essential elements in the leaves and berries of four *Vaccinium* species (*Vaccinium corymbosum* L., *Vaccinium myrtillus* L., *Vaccinium macrocarpon* Ait., *Vaccinium oxycoccos* L.). The levels of Ca, Mg, iron (Fe), copper (Cu), zinc (Zn) and manganese (Mn) were estimated by atomic absorption spectrophotometer, content of N, P, molybdenum (Mo) and boron (B) by colorimetry, sulphur (S) by turbidimetry and K by a flame photometer with air propane-butane flame. Faiku et al. (2022) used atomic absorption spectroscopy, too, for the determination of eight elements, Ca, Na, Mg, Cu, Fe, Mn, Zn and chromium (Cr) in the tea leaf samples of *Vaccinium macrocarpon* the tea infusions. The number of papers about using of SEM–EDX in this way is quite limited.

The determination of the elemental composition of plant material can be useful for the qualitative determination of food, dietary supplements or medicinal herbs. The SEM–EDX can reveal the presence of toxic elements or indicates high concentrations of macro- and micronutrients. In addition, its great advantage is the direct analysis of the material without its special pre-processing associated with time requirements (Filipiak-Szok et al., 2015).

In the present work, the SEM–EDX and high pressure liquid chromatography with diode array detection (HPLC–DAD) methods were used for achieving the main object to evaluate the environmental pollution and its influence on the chemical composition and phytochemical quality of the leaves of *Vaccinium* sp. plants growing in the mine-polluted and control area. SEM–EDX provided elemental identification and quantitative compositional information of plant leaves and soils from localities where plants grew.

HPLC–DAD has provided insight into the presence and content of major antioxidants. The use of these methods would represent a rapid and efficient way to assess environmental pollution and its impact on the phytochemical quality of plants.

## Material and methods

### Plant material

The leafy above-ground parts of *Vaccinium* sp. without flower buds and flowers were collected in the locality close to the iron ore mine near Smolník village (560 m a.s.l.) and in the control locality called Zlatý stôl (1322 m a.s.l). Both localities differ in mutual elevation; therefore, the control site is minimally impacted by the mining and industrial activity.

The cuttings were carefully saved in zip-sealed plastic bags stored in portable refrigerated plastic box and transferred to the laboratory. Eight cuttings from each tested group were watered through the transport and used for instant analysis of the fluorescence of chlorophyll* a* and the size of the leaf area. Cuttings for elemental and phytochemical analyses were air-dried in the dark room at laboratory temperature for 5 days. Then, they were stored in the zip-sealed plastic bags in the fridge (4 °C). The green colour of dried leaves indicates adequate quality of the leaf drug (Kašpárová, 2009). Identification of plant material was carried out by comparing the samples with specimen stored in the herbarium of the Botanical Garden of Pavol Jozef Šafárik University. For elemental and phytochemical analyses, dried leaves were taken from the leafy dried twigs. The leaves for elemental analyses were stored in plastic tubes and processed according to the requirements of SEM–EDX method (described below). The leaves for phytochemical analyses were homogenised and extracted by 80% methanol, subsequently centrifuged and stored in the fridge at 4 °C.

### Determination of environmental quality

The elemental analyses and pH determinations of soils taken from the chosen areas were carried out for assessment of the environmental quality. Samples were taken from the O-layer of five different sites within the location where plants were growing. They were stored in the 50-ml centrifuged tubes. One hundred grams of each soil sample was extracted by 100 ml of deionised water (Millipore Direct-Q 3UV with pump, Merck, Darmstadt, Germany). Subsequently, soil solution was filtrated and centrifuged (4500 × min^−1^). Its pH was evaluated by a manual pH meter (Agilent 3200P pH meter, Santa Clara, CA, USA).

### Qualitative and quantitative analysis of chemical elements by SEM–EDX

Elemental analysis of leaf and soil samples was measured by a JEOL JSM IT 300 scanning electron microscope (SEM) equipped with an EDAX system (Ametek GmbH, Weiterstadt, Germany) for performing energy-dispersive X-ray microanalysis (EDX) (Sassmann et al., 2015). Samples for elemental analyses were dried and carbon coated in a 5–10-nm carbon layer (Leica; Carbon coater, MED 020, Vienna, Austria) to prevent surface charge. The leaves and soil samples were mounted on 0.5″ aluminium specimen stubs covered with SEM-carbon foils (PELCO Tabs™ Carbon Conductive Tabs, Double Coated, Christine Gröpl, Austria). For specific spectra analyses, background subtraction and data collection, the EDAX-TEAM software version V4.3 (Ametek Material Analysis, Berwyn, Pennsylvania) was used. For deconvolution of the spectra, corrections for interference between elements were applied according to the software. Elemental analyses were performed with the following constant SEM settings: acceleration voltage of 20 kV, working distance 11 mm (sample to the final lens), take-off angle 35.1, dead time 30% and measurement time of 50 s (Lsec 50) for each measurement. From each sample, multiple measurements (*n* = 10) were taken. Taking into account that samples show uneven surface and texture, all element analyses were performed at a magnification of 500 × –750 × for detected material. Attention was taken to the orientation of sample surface in respect to the silicium drift detector (SDD), model Octane Plus Det (Ametek GmbH, Weiterstadt, Germany); geometry was kept stable. Thereby, a possible blur of the measurements could be minimalised.

As EDX is a semiquantitative method and the content of selected elements is detected in relation to all other elements, only the following elements were measured: C, N, O, Na, Mg, Al, Si, P, S, Cl, K, Ca, Mn, Fe and Cu. Other metals were under the detection limit of the method. All contents are shown as weight percent of the dry mass (wt %).

### Determinations of total soluble phenols, flavonoids and anthocyanins content

Total soluble phenols (TP) and flavonoids (TF) were determined spectrophotometrically (Synergy HT, Biotek, Hampton, USA) from methanolic extract of dry leaves (0.1 g DW to 1 ml of 80% methanol). TP content was determined at 750 nm using the Folin-Ciocalteu method (Dučaiová et al., 2016) with gallic acid as standard (Sigma-Aldrich). For evaluation of TF content, the aluminium chloride method working with 420 nm wavelength and quercetin (Sigma-Aldrich) as standard were used (Ordoñez et al., 2006).

The content of total anthocyanins was determined spectrophotometrically at 530 nm and 657 nm using a spectrophotometer (Jenway 7310 Advanced visible spectrophotometer, Cambridgeshire, UK) (Neff & Chory, 1998). After homogenisation of 50 mg of dry leaves, 2 mL of acid methanol I [1% HCl in MeOH (v/v)], 1.4 mL of deionised water and 3.5 mL chloroform were added. The mixture was centrifuged at 10,000 rpm for 5 min. Subsequently, 2.5 mL of supernatant was supplemented to a final volume of 3 mL with acid methanol II (60% of acid methanol I: 40% of deionised water) and was analysed spectrophotometrically.

### Determination of antioxidant activity

DPPH assay is based on the reaction of purple-coloured free radicals with the scavenger to yield the colourless product 1,1-diphenyl-2-picrylhydrazine. One hundred microlitres of 80% methanolic extract from *Vaccinium* sp. leaves was added to 900 µl of 0.2 mmol.l^−1^ methanol DPPH solution. Incubation has been running for 30 min at 25 °C in the dark. DPPH assay was realised in eight repetitions for both the control and the mine-influenced plants of both species. Antioxidant activity was expressed as a percentage of reduction of DPPH radicals (Popović et al., 2012).

### Determination of fluorescence of chlorophyll *a* and the size of the leaf area

FluorCam 800 MF (Photon Systems Instruments Ltd, Brno, Czech Republic) was used for the evaluation of the fluorescence of chlorophyll* a* (chl *a*) and the size of leaf area on the fresh *Vaccinium* sp. leaves taken from the water saturation cutting instantly after the transport from the locality. Measurements were performed after illuminating the dark-silenced leaves with saturation flash (2000 µmol^−1^ m^−2^) for 1 s. The fluorescence of chlorophyll *a* was determined as the ratio of variable fluorescence (F_V_) and maximal fluorescence (F_M_). The leaf areas were measured simultaneously with the fluorescence of chl *a*. (Paľove-Balang et al., 2015).

### HPLC–DAD determination of main *Vaccinium* sp. phytochemicals

Main secondary metabolites accumulated in the leaves of *V. myrtillus* and *V. vitis-idaea* (chlorogenic acid, hyperoside, isoquercetin, rutin, arbutin and hydroquinone) were confirmed with the use of purchased commercial standards: chlorogenic acid (purity ≥ 95%, Fluka, Busch, Switzerland); hyperoside (purity ≥ 95%, Roth, Germany); isoquercetin (purity ≥ 95%, Roth, Germany); and all other, such as rutin (purity ≥ 95%), arbutin (≥ 98%) and hydroquinone (pharmaceutical secondary standard) which were purchased from Sigma-Aldrich (Missouri, USA). Methanol and acetonitrile for HPLC were purchased from Sigma-Aldrich too.

Twenty microlitres of leaf methanolic extract (50 mg DW to 2.5 mL 80% methanol) were used for HPLC–DAD (Agilent Technologies 1260 Infinity, Santa Clara, CA, USA) analyses in three repetitions. Chromatographic equipment consisted of degasser, autosampler and binary pump and DAD detector. Separation of methanolic extracts was realised through the Kromasil 100 C18 (5 µm, 250 × 4.6 mm, Göteborg, Sweden), with mobile phases A (5% acetonitrile with 5% of trifluoracetic acid) and B (80% acetonitrile) in gradient program: 0 min (A 90%); 25 min (A 50%); 35 min (A 0%); 45 min (A 0%); and 50 min (90%), with a flow rate of 0.7 ml min^−1^. The chosen wavelengths for detection of metabolites were for chlorogenic acid 320 nm, for flavonoids 350 nm and for arbutin and hydroquinone 220 nm. For the evaluation of obtained chromatograms, the software ChemStation rev. B.04.03 [16] (Agilent, Santa Clara, CA, USA) was used.

From HPLC–DAD chromatograms, main secondary metabolites were determined based on agreement in chosen features (retention times, absorption maxims, shape of UV spectral curves) using commercial standards. For the construction of calibration curves of detected metabolites, four different volumes (5, 10, 15 and 20 µl) in three repetitions were taken from stored standard solutions.

### Statistical analysis

The obtained data were statistically analysed using ANOVA and Tukey’s test in Minitab Release 11 (Minitab Inc., State College, PA, USA) and by Student’s *t*-test realised through the PAST (PAleontological STatistics, version 3.10, 1999–2015). The (n) in the figures denotes the number of measured replications. Different letters above columns indicate statistically significant differences between controls and plants from mining locality. Small letters belong to *V. myrtillus* plants and large letters belong to *V. vitis-idaea* plants.

## Results and discussion

Dry leaves of *Vaccinium* sp. plants are used as medicinal drugs, so the determination of their elemental composition and the content of active phytochemicals (e.g. flavonoids, anthocyanins, phenylpropanoids, quinones) should participate in the evaluation of their pharmaceutical or nutrient quality (Kandziora-Ciupa et al., 2017; Zhang et al., 2023). The drying conditions, including temperature, significantly impact the composition of the medicinal drug. The air-drying at the laboratory temperature or at a maximum temperature of 40 °C is optimal to preserve the phytochemical content. Moreover, during the harvesting of the leaves, these cannot be damaged, because through the drying process, they turn brown and their quality decreases (Kašpárová, 2009). This is due to disruption of the leaf cell structure and dissociation of polyphenols and iridoids. After that, contact between iridoids and *ß*-glucosidase or polyphenols and polyphenol oxidases is much easier and the oxidation of polyphenols or catalysis of iridoids is induced (García-Rodríguez et al., 2011; Murata et al., 1995). Application of higher temperatures has a destructive effect on enzyme activity, so the content of phenolics significantly changes. Miao et al. (2020) studied different ways of the *Vaccinium bracteatum* T. leaves drying and showed their impact on the physicochemical properties of dried leaves. They found that direct drying at 60 °C reduced the phenolic content by more than 67.6% and 76.9% for iridoids. The activity of related enzymes decreased to minimal. Similarly, the antioxidant power of air-drying leaves was markedly lower than that of the fresh leaves. Lower drying temperatures preserve enzyme activity for a longer time and allow for the gradual release of substances from inactive forms. Because of that, the drying at laboratory temperature is adequate for optimal quality of the medicinal drug.

*Vaccinium* sp. plants are able to tolerate various pollutions thanks to the accumulation and processing of chemical elements like Mn, Cu or Pb (Białońska et al., 2006; Brekken & Steinnes, 2004; Mróz & Demczuk, 2010; Prodaj & Kompišová Ballová, 2021). Because of that, they can grow close to mining areas, like the locality of the Smolník mines. The reason for the selection of this site in our work was to verify the bioaccumulation of elements in plants *Vaccinium* sp. from a polluted area with an excess of macro- and micronutrients and to compare it with the bioaccumulation of plants from an unpolluted site.

The control locality is approximately 24 km away from the mine site and its elevation is almost twice that of the mine’s locality. The pollution produced by the mine activity has not impacted the soil composition of the control site. Our data from the elemental analyses of the soils has shown differences in the content of the metals and macronutrients (Table 1). The soil from the vicinity of the mine has had an excess of N, O, Na, P, Mg, S, Cl, Ca, Mn, Fe and Cu. Our data largely follow the results of studies dealing with distribution of metals in the Smolník area published earlier (Balintova et al., 2012; Banásová, 1983; Jaško et al., 1998), which confirmed the pollution caused by mining activity in this locality.

**Table 1:** SEM–EDX qualitative and quantitative analyses of soils and *Vaccinium sp*. leaves from control and the mine-influenced locality (MIL). Data are presented as weight percent of the dry mass [wt %] (*n* = 10; *P < 0,05; **P < 0,01; ***P < 0,001; dash means without significant difference)

Element	*Soil*			*V. myrtillus*			*V. vitis-idaea*		
	Control	MIL		Control	MIL		Control	MIL	
***C***	25.061 ± 0.739	14.772 ± 0.423	***	72.268 ± 0.733	65.507 ± 1.167	***	40.386 ± 0.874	58.628 ± 1.753	***
***N***	3.986 ± 0.329	4.766 ± 0.242	**	3.775 ± 0.220	2.968 ± 0.248	***	7.432 ± 0.428	7.434 ± 0.385	-
***O***	43.142 ± 2.481	51.082 ± 4.586	***	22.201 ± 0.789	28.677 ± 1.333	***	48.446 ± 1.202	31.912 ± 2.583	***
***Na***	0.569 ± 0.122	2.019 ± 0.643	***	0.016 ± 0.013	0.046 ± 0.027	*	0.075 ± 0.025	0.049 ± 0.043	-
***Mg***	0.544 ± 0.088	0.847 ± 0.014	*	0.214 ± 0.036	0.375 ± 0.052	***	0.473 ± 0.037	0.179 ± 0.028	***
***Al***	4.163 ± 0.978	4.022 ± 0.953	-	0.052 ± 0.018	0.049 ± 0.023	-	0.068 ± 0.010	0.059 ± 0.022	-
***Si***	18.927 ± 1.134	14.574 ± 2.593	***	0.078 ± 0.026	0.046 ± 0.019	*	0.081 ± 0.036	0.072 ± 0.039	-
***P***	0.157 ± 0.083	0.218 ± 0.012	-	0.098 ± 0.015	0.092 ± 0.010	-	0.196 ± 0.299	0.112 ± 0.039	**
***S***	0.063 ± 0.009	0.738 ± 0.092	***	0.103 ± 0.013	0.211 ± 0.014	***	0.187 ± 0.021	0.152 ± 0.044	-
***Cl***	0.011 ± 0.001	0.113 ± 0.018	-	0.066 ± 0.022	0.179 ± 0.043	**	0.158 ± 0.013	0.039 ± 0.017	***
***K***	1.577 ± 0.323	0.983 ± 0.004	**	0.432 ± 0.095	0.817 ± 0.097	***	1.907 ± 0.149	0.632 ± 0.261	***
***Ca***	0.033 ± 0.002	0.277 ± 0.069	*	0.467 ± 0.148	0.774 ± 0.062	***	0.876 ± 0.034	0.379 ± 0.084	***
***Mn***	0.056 ± 0.003	0.268 ± 0.048	*	0.076 ± 0.015	0.094 ± 0.021	-	0.201 ± 0.027	0.109 ± 0.048	***
***Fe***	1.033 ± 0.022	5.013 ± 0.844	***	0.064 ± 0.018	0.068 ± 0.014	-	0.092 ± 0.013	0.103 ± 0.028	-
***Cu***	0.221 ± 0.034	0.337 ± 0.078	-	0.089 ± 0.014	0.072 ± 0.017	-	0.127 ± 0.015	0.151 ± 0.027	*

From the visual evaluation of chosen areas, soil from the slag heap close to the mining entrance has had rusty-brown or metallic grey shade compared to the blackish-brown soil from the control locality. The colour of soil depends in large range on its pH and metal representation. From our measurements, pH of metallic grey soil was 3.61, for the rusty-brown 2.88 and for the control 5.1. The ochre colouring of soil is made up of the process of oxidation on the surface. Balintova et al. (2012) have proved the progressive oxidation of Fe^2+^ ions to Fe^3+^ by oxygen from air and its precipitation to the form of Fe(OH)_3_ continually with growing environment pH.

**Fig. 1:**
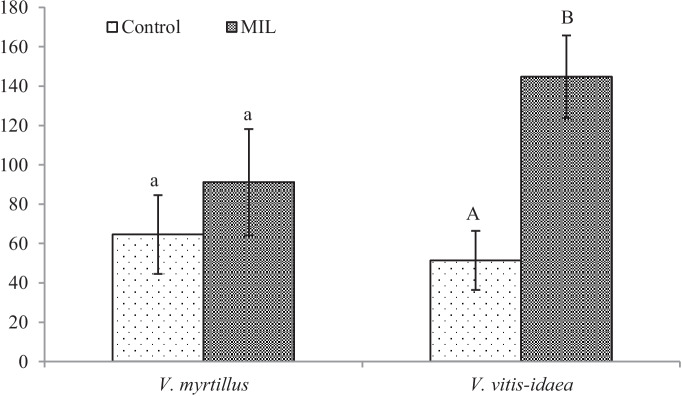
Leaf area of *Vaccinium* sp. plants from control and mining locality [mm^2^] from the control and the mine-influenced localities (MIL). Data are means ± SDs (*n* = 8). Values within column, followed by the same small or large letter(s), are not significantly different according to Tukey’s test (*p* < 0.05)

Some chemical elements mentioned above are used by *Vaccinium* plants as nutrients and can positively influence the growth of leaves at higher altitudes. Results of the paper of Prodaj and Kompišová Ballová (2021) have proved that the accumulation of elements in the plant parts of *V. myrtillus* depends on the elevation. From their results, S, Cl, K, Ca, Cr, Fe and Ba have been accumulated in leaves more significantly at higher altitudes and heavy metals at lower altitudes. This could be the reason for better plant growth in the higher situated areas. Previously publicated papers pointed out to positive impact of elevation gradient on the growth of *V. myrtillus* leaves (Pato & Obeso 2012). The leaf area, length and width of leaves and simultaneously total content of chlorophyll are in positive correlation with increasing altitude. Yüksek et al. (2013) obtained similar results in work with similar aims but realised on *Vaccinium arctostaphylos* L. The authors subdivided the research area into three altitude groups and found out that the leaf width was decreasing according to altitude; at plants growing around 1300 m.a.s., the medium length and the highest leaf area were recorded. However, our results showed a larger leaf area in the MIL group growing in the lower situated site (Fig. 1), which can be related to the availability of mineral nutrients (Glonek & Komosa, 2013).

**Fig. 2:**
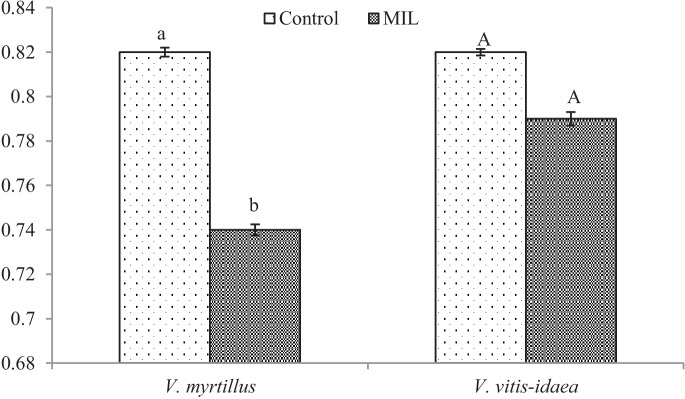
Fluorescence of chlorophyll *a* (FV/FM) at *Vaccinium* sp. plants from the control and the mine-influenced localities (MIL). Data are means ± SDs (*n* = 8). Values within column, followed by the same small or large letter(s), are not significantly different according to Tukey’s test (*p* < 0.05)

Despite better leaf growth in the MIL groups, a fluorescence of chlorophyll *a* in these leaves decreased (Fig. 2). The fluorescence of chlorophyll *a* reflects the effectiveness of photosynthetic apparatus and is expressed as a ratio of variable and maximal fluorescence (F_variable_/F_maximal_). For higher plants, optimal fluorescence values are in the range from 0.75 to 0.85. Values lower than 0.75 indicate the dysfunctionality of photosynthetic apparatus (Murchie & Lawson, 2013). Our measurements have shown that control leaves of *V. myrtillus* have fully functioning photosynthetic apparatus; however, the MIL leaves have none. A similar situation was recorded for the leaves of *V. vitis-idaea* (Fig. 2), but without significant differences. The reason for the drop in the fluorescence values in the MIL group can lie in the composition of the soil again. An excess of metals, such as Cu, Ni, Hg, Cd or Zn, in the soil or in the plant tissues can decrease the quantum efficiency of photosystem II due to their exchange with central chlorophyll Mg ions (Żurek et al., 2014; Zheng et al., 2019; Jaghdani et al., 2020). The content of Mg in our tested leaves groups varied, similarly content of Fe or Cu, too, so changes in the composition of chlorophyll *a* and in its activity were possible.

SEM–EDX analyses of *Vaccinium* sp. leaves from the control site revealed different strategies of the elements processing depending on the species. *V. vitis-idaea* controls showed a higher bioaccumulation capacity compared to controls of *V. myrtillus*. In addition, data has shown comparable levels of Al, Si, P and Fe for both control groups, so in the case of these elements, the similar processing strategy and utilisation can be assumed. However, it is necessary to remember the fact that *V. vitis-idaea* is non-deciduous, and the levels of determined elements are accumulated lifetime. Despite of morphological similarity of the chosen species and their botanical classification, their ways of survival in the polluted environment differ. Kandziora-Ciupa et al. (2017) directly revealed different reactions of *Vaccinium* sp. plants induced by harmful environment. They found differences in the bioaccumulation of heavy metals and the biochemical processes. An enhanced accumulation of Mn and more activated non-enzymatic antioxidant system were more typical for the *V. myrtillus* plants and higher concentrations of heavy metals and a more activated enzymatic antioxidant system were more significant in the *V. vitis-idaea* plants.

From our next data, the effect of pollution on *Vaccinium* leaves is expressive. In the MIL groups, mutually opposite accumulations between species were found. In the leaves of *V. myrtillus*, increase of elemental content has predominated, except for C, N, Al, Si, P and Cu, but in the *V. vitis-idaea*, elemental content decreased, except for C, Fe and Cu. Their levels were enhanced, even significantly in the case of Cu. Later published papers claimed that the main bioaccumulation of metals is in the roots and their transport from roots to shoots is restricted (Kandziora-Ciupa et al., 2017; Stefanowicz et al., 2016), but the long-lasting effect of environmental excess of metals and their lifetime accumulation by plants have participated on their high leaf concentrations.

The intensity of elemental accumulation depends on soil pH, and in the case of heavy metals, a low pH allows their uptake by the roots (Kula et al., 2018).

As can be seen, leaves of *V. myrtillus* did not accumulate metals primarily. Based on the results of the work of Stefanowicz et al. (2016), we assumed their significant presence in the roots with limited transport to the other above-ground parts of the plant.

Mn is found in the leaves of *V. myrtillus* often in concentrations above the limits and it is the reason why this plant is considered its hyperaccumulator (Kandziora-Ciupa et al., 2017; Mróz & Demczuk, 2010). Leaves of both our control groups contained more Mn amount than that which was present in the soil. In the conditions of pollution, with higher Mn content, the leaves of *V. myrtillus* stored more Mn than in the controls; however, leaves of *V. vitis-idaea* do not.

The content of Na, Cl and Ca is variable depending on the quality of the environment. Their higher abundance in the polluted soil was reflected in the foliar content of *V. myrtillus*. In the case of *V. vitis-idaea*, the content of all mentioned compounds decreased significantly. A similar situation has been shown after fertilisation of *Vaccinium* plants in field conditions, where an increased uptake of these elements is occurring (Bryla et al., 2012).

In the case of K, its decreased level in the polluted soil did not reflect its 88% increment in the *V. myrtillus* leaves, but vice-versa, its foliar content in the *V. vitis-idaea* correlated with K content in the soil.

Due to similarities in physicochemical properties between Na^+^ and K^+^, Na^+^ could compete with K^+^ for major binding sites in key metabolic processes (e.g. Na^+^ competes with K^+^ for uptake sites at the plasma membrane) and cause disturbances in plant metabolism. High concentration of Na^+^ in the soil solution (such as is our MIL soil) reduced K^+^ availability, which can lead to disintegration of plasma membrane and may promote K^+^ leakage, resulting in a rapid decline in cytosolic K^+^(Wang et al., 2013). From our results, content of Na and Cl in the MIL soil increased, but content of K in the MIL *V. myrtillus* leaves increased too. So, its uptake was not influenced by high concentration of Na and Cl. However, this may be the reason for lower K content in the *V. vitis-idaea*. In general, higher K^+^ accumulation in plants is considered characteristic features of stress-tolerant plants (like *V. myrtillus*) and keeping of cellular K^+^ content above a certain threshold and maintaining a high cytosolic K^+^/Na^+^ ratio is critical for plant growth (Shabala, 2017; Wang et al., 2013).

Silicates are forms of Si entering the plant roots and transported to above-ground plant parts, where they polymerise and create intracellular silica bodies. Experiments dealing with the removal of Si from media containing its different concentrations have shown a positive correlation between its content in the medium and in the tissues. A high concentration of Si in the medium enhances the concentration of Si in the plant parts (Nakphlaiphan et al., 2016; Rossini Oliva et al., 2009). Previous publicated paper has shown the accumulation of Si in *V. myrtillus* leaves in the form of phytoliths, which occur in several parts of the leaf (e.g. the palisade mesophylls of the upper epidermis, or in the lower epidermis around the stomata, including guard cells) (Morikawa & Saigusa, 2004). In our case, a lower Si level was recorded, which corresponded with the reduced Si level in mine-polluted soil.

Comparing obtained SEM–EDX quantitative data with other previously published data is challenging due to the lack of similar papers or using of different methods (Dróżdż et al., 2017; Faiku et al., 2022; Filipiak-Szok et al., 2015; Mróz & Demczuk, 2010; Reddy et al., 2024). For example, Karlson et al. (2018) determined six elements by atomic absorption spectrophotometer, and other six elements by colorimetry, turbidimetry and flame photometry. *European Pharmacopoeia* does not classify an optimal chemical composition of *Vaccinium* leaf drugs, nor optimal methods for elemental determinations. Moreover, the European Union legislation does not define the methods and qualitative requirements for optimal chemical composition of dietary supplements or medicinal plant drugs, except for Pb, Cd, Hg, inorganic tin and arsenic (Comission Regulation (EU), No. 915, 2023). Therefore, commercially available medicinal or dietary supplements do not contain information about the limits of toxic element concentrations. From this point of view, SEM–EDX may be used as an effective and rapid method for the determination of the element complex that makes up the analysed material without demanding sample pre-processing.

Another part of our work was to evaluate the production of phytochemicals in plants growing in two qualitatively different environments. Environmental pollution (such as the presence of toxic elements or excess nutrient elements) is often the reason for increased production of secondary metabolites, precisely those with high antioxidant potential. Excess of elements such as S, Mn, Fe and Cu increases the intensity of secondary metabolism by activating metabolic enzymes, which has been recently published (Zhang et al., 2023). Higher levels of phenol-related compounds are one of the plant reactions induced by environmental pollution, typically in the case of *Vaccinium* sp. plants.

From our analyses of phenol-related compounds, for *V. myrtillus* plants, only the content of total flavonoids (Fig. 4) increased significantly. Interestingly, anthocyanins (Fig. 5) were confirmed in the green leaves obtained from the polluted locality, which is more shady, without direct sunlight. As was published previously (Riihinen et al., 2008), the green leaves of *V. myrtillus* growing in normal shadow conditions did not contain anthocyanins. Therefore, the production of anthocyanins in green leaves from the mine-influenced area may be due to the effect of pollution. A minimal amount of anthocyanins was found in the control leaves, which were growing in the site at higher altitude. Their production could be induced by effect of the factors like higher amount of UV and sunlight.

**Fig. 3:**
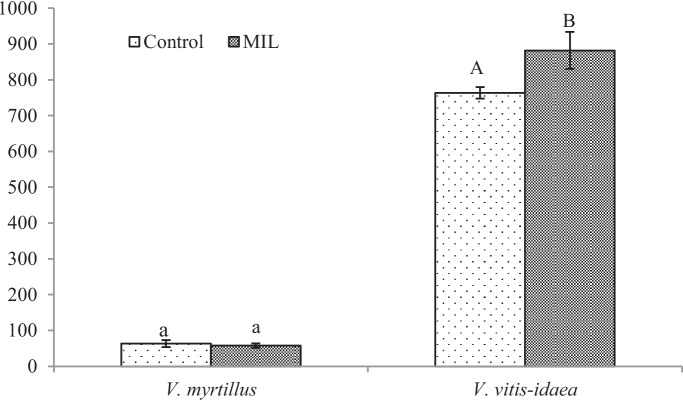
Content of total soluble phenols [mg g.^−1^ DW]. Data are means ± SDs (*n* = 8). Values within column, followed by the same small or large letter(s), are not significantly different according to Tukey’s test (*p* < 0.05)

**Fig. 4:**
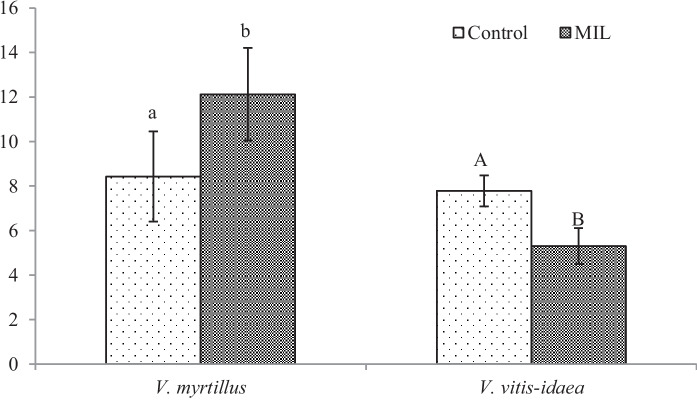
Total flavonoids content [mg g.^−1^ DW]. Data are means ± SDs (*n* = 8). Values within column, followed by the same small or large letter(s), are not significantly different according to Tukey’s test (*p* < 0.05)

**Fig. 5:**
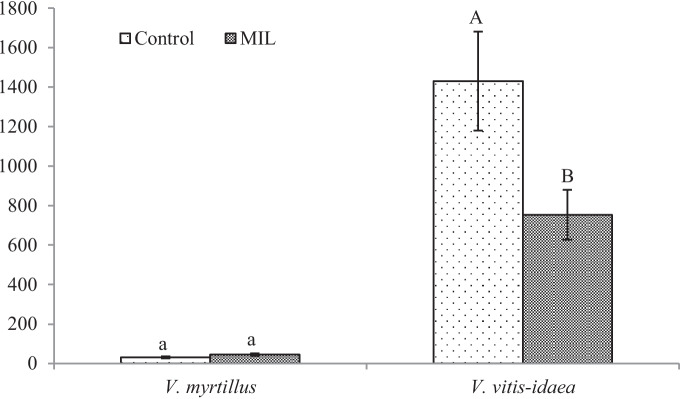
Content of total anthocyanins [mg g.^−1^ DW]. Data are means ± SDs (*n* = 8). Values within column, followed by the same small or large letter(s), are not significantly different according to Tukey’s test (*p* < 0.05)

For MIL *V. vitis-idaea* leaves, the content of total soluble phenols (Fig. 3) increased significantly, but a significant decrease for total flavonoids (Fig. 4) and anthocyanins (Fig. 5) was observed.

In addition to the fact that mine pollution affects the production of phytochemicals in the leaves, we must also consider the effect of altitude on the production of antioxidants, which was indicated above. As we mentioned earlier, our control locality has almost twice the elevation of the mine area. The vegetation growing at high altitudes is exposed to more intensive ultraviolet radiation (UV), precipitation or changes in temperatures. Most of the papers investigating the effect of higher UV radiation on *Vaccinium* plants deal with its effect to fruit ripening (e.g. Li et al., 2021; Yang et al., 2019) and works realised on the leaves dealing with the production of metabolites are missing. Since phenol-related compounds, including flavonoids, phenylpropanoids and anthocyanins, have a substantial antioxidative effect, their tissue concentration increases after exposure to higher UV, even after short-term effect (Petruľová et al., 2014; Petruľová et al., 2020). Based on the mentioned, we deduced lower leaf production of phenol related compounds in the leaves from lower altitudes. For therapeutical application, leaves with higher phenolic production are more suitable, so we have finally decided to choose a control locality with a higher altitude. Nevertheless, the effect of soil pollution on the production of phytochemicals in all MIL leaves is so significant that exceeds effect of factors of higher altitude, mainly in the case of flavonoids and anthocyanins in MIL *V. myrtillus* and total soluble phenols of the MIL *V. vitis-idaea*. The high content of flavonoids and anthocyanins in the latest mentioned group can be related to the impact of high altitude factors.

For a more precise evaluation of pollution’s impact on the phytochemical production of two *Vaccinium* species, we selected six major metabolites produced in the leaves of the *Vaccinium* sp. All of them have already been characterised in previously realised works using a combination of analytical approaches, like liquid chromatography and ^1^HNMR (Durazzo et al., 2019; Ek et al., 2006; Liu et al., 2014; Wu et al., 2019). Chlorogenic acid (dominant in *V. myrtillus*), arbutin and hydroquinone (dominant in *V. vitis-idaea*) and flavonol glycosides (hyperoside, isoquercetin, rutin) were chosen for to achieve our goal evaluating.

Verification of metabolic identity in our chosen *Vaccinium* species was carried out by comparison of their UV spectra with typical absorption maxima (around 220, 320 and 350 nm), as well as their retention times with those of the reference compounds obtained commercially. For *V. myrtillus* leaves, significant differences were recorded for flavonoids, such as hyperoside and isoquercetin, and minimal for chlorogenic acid, arbutin and hydroquinone (Table 2).

**Table 2:** Content of main phytochemicals in the leaves of *Vaccinium sp*. plants [mg/g DW] from control and the mine-influenced locality (MIL). Data are means ± SDs (*n* = 8). Values within column, followed by the same small or large letter(s), are not significantly different according to Tukey’s test (*P* < 0.05)

	*Chlorogenic acid*	*Hyperoside*	*Isoquercetin*	*Rutin*	*Arbutin*	*Hydroquinone*
*V. myrtillus*						
Control	66.269 ± 17.243	0.999 ± 0.513a	13.854 ± 3.265 a	6.347 ± 0.067	1.752 ± 0.315	0.796 ± 0.130 a
MIL	68.502 ± 12.501	1.407 ± 0.464b	22.916 ± 4.901 b	6.364 ± 0.022	1.464 ± 0.328	0.999 ± 0.177 b
*V. vitis-idaea*						
Control	0.475 ± 0.131 A	0.254 ± 0.112 A	1.812 ± 0.766 A	1.906 ± 0.088	14.171 ± 2.356 A	4.272 ± 0.755
MIL	0.799 ± 0.184 B	0.016 ± 0.001 B	0.171 ± 0.029 B	1.861 ± 0.049	18.953 ± 3.844 B	5.271 ± 0.854

For *V. vitis-idaea* leaves, increases in chlorogenic acid, arbutin and hydroquinone production were observed, while the levels of flavonoids (hyperoside and isoquercetin) dropped to a detectable minimum (Table 2). Our spectrometrical data dealing with total soluble phenols, flavonoids and anthocyanins were consistent with those obtained by HPLC–DAD.

**Fig. 6:**
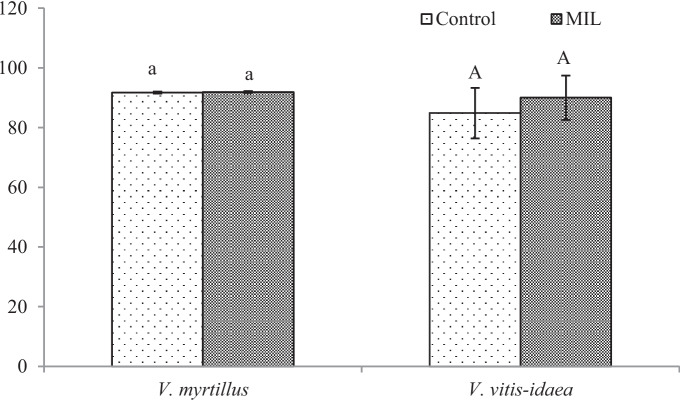
Antioxidant activity [% inhibition]. Data are means ± SDs (*n* = 8). Values within column, followed by the same small or large letter(s), are not significantly different according to Tukey’s test (*p* < 0.05)

Changes in the metabolic levels had an impact on the antioxidant activities of alcohol *Vaccinium* leaf extracts, which were established by DPPH radical assay. Every extract from the control and the pollution-influenced leaves has proved a high rate of scavenging of DPPH radicals, which is consistent with the confirmed production of metabolites with antioxidative potential (Fig. 6). In the case of *V. myrtillus* plants, the minimal differences in antioxidant activity were recorded between control and pollution-affected plants due to minimal differences in the production of chlorogenic acid and hydroquinone. In addition, higher levels of inactive glucoside form of hyperoside and isoquercetin did not contribute to the antioxidant capacity of extract from the mine-influenced leaves.

For the antioxidant activity of *V. vitis-idaea* extracts, increased levels of chlorogenic acid and hydroquinone declared increased of antioxidant activity of extract from the mine-polluted leaves, while low levels of hyperoside and isoquercetin did not influence it. Our results are in agreement with the one published earlier, which proved a strong correlation between the antioxidant activity of *Vaccinium* sp. leaves and the content of phenols (Bujor et al., 2019).

## Conclusions

In summary, our paper offers insight into the bioaccumulation of elements and phytochemical production of the leaves of two medicinal plants, *V. myrtillus* L. and *V. vitis-idaea* L., growing in qualitatively different environments. The aim of our paper was to detect the presence of toxic elements or their high concentrations in the *Vaccinium* leaf drugs and to evaluate impact of polluted environment on the leaf phytochemical production. These species are used in environmental quality biomonitoring or as a popular medicinal drug. Actual legislative documents, dealing with the nutrient quality of food, determine limits only for Pb, Hg, Cd, As or Sn, and information about other elements such as Cu, S, Al or Mn, is missing, despite the fact that the high concentration of micro- or macronutrients in the tissues may be harmful not only for plants but also for future consumers too. Based on these statements, questions about the suitable concentration limits of elements or methods of their determination to characterise the quality of plants appointed as medicinal plant drugs or dietary supplements are arising. A combination of SEM–EDX and HPLC–DAD methods may be a suggestion for a rapid and effective way of determining mentioned limits with the aim to characterise plant material in more detail.

## Data Availability

No datasets were generated or analysed during the current study.
